# Development of Antibody-Modified Nanobubbles Using Fc-Region-Binding Polypeptides for Ultrasound Imaging

**DOI:** 10.3390/pharmaceutics11060283

**Published:** 2019-06-15

**Authors:** Nobuhito Hamano, Sho Kamoshida, Yamato Kikkawa, Yusuke Yano, Tomomi Kobayashi, Yoko Endo-Takahashi, Ryo Suzuki, Kazuo Maruyama, Yuji Ito, Motoyoshi Nomizu, Yoichi Negishi

**Affiliations:** 1 Department of Drug Delivery and Molecular Biopharmaceutics, School of Pharmacy, Tokyo University of Pharmacy and Life Sciences, 1432-1 Horinouchi, Hachioji, Tokyo 192-0392, Japan; nhamano@toyaku.ac.jp (N.H.); y131078@toyaku.ac.jp (S.K.); y141191@toyaku.ac.jp (Y.Y.); y154098@toyaku.ac.jp (T.K.); endo@toyaku.ac.jp (Y.E.-T.); 2 Department of Clinical Biochemistry, School of Pharmacy, Tokyo University of Pharmacy and Life Sciences, Tokyo 192-0392, Japan; kikkawa@toyaku.ac.jp (Y.K.); nomizu@toyaku.ac.jp (M.N.); 3 Laboratory of Drug and Gene Delivery Research, Faculty of Pharma-Sciences, Teikyo University, 2-11-1 Kaga, Itabashi-ku, Tokyo 173-8605, Japan; r-suzuki@pharm.teikyo-u.ac.jp (R.S.); maruyama@pharm.teikyo-u.ac.jp (K.M.); 4 Graduate School of Science and Engineering, Kagoshima University, 1-21-35 Korimoto, Kagoshima 890-0065, Japan; k2174603@kadai.jp

**Keywords:** antibody-modified nanoparticles, nanobubbles, Fc-region-binding polypeptide, ultrasound tumor imaging

## Abstract

Ultrasound (US) imaging is a widely used imaging technique. The use of US contrast agents such as microbubbles, which consist of phospholipids and are filled with perfluorocarbon gases, has become an indispensable component of clinical US imaging, while molecular US imaging has recently attracted significant attention in combination with efficient diagnostics. The avidin–biotin interaction method is frequently used to tether antibodies to microbubbles, leading to the development of a molecular targeting US imaging agent. However, avidin still has limitations such as immunogenicity. We previously reported that lipid-based nanobubbles (NBs) containing perfluorocarbon gas are suitable for US imaging and gene delivery. In this paper, we report on the development of a novel antibody modification method for NBs using Fc-region-binding polypeptides derived from protein A/G. First, we prepared anti-CD146 antibody-modified NBs using this polypeptide, resulting in high levels of attachment to human umbilical vein endothelial cells expressing CD146. To examine their targeting ability and US imaging capability, the NBs were administered to tumor-bearing mice. The contrast imaging of antibody-modified NBs was shown to be prolonged compared with that of non-labeled NBs. Thus, this antibody modification method using an Fc-binding polypeptide may be a feasible tool for developing a next-generation antibody-modified US imaging agent.

## 1. Introduction

Ultrasound (US) imaging is a frequently used diagnostic technique that offers high spatial resolution, allows for real-time imaging, and combines the advantages of non-invasiveness without the use of ionizing radiation and at a low cost [1,2]. US contrast agents are gas-filled, echogenic microbubbles that remain exclusively in the vascular compartment [2]. The application of microbubbles has become an indispensable component of clinical US imaging [3], and molecular imaging via US has recently been reported [4]. Microbubbles aid in enhancing the specificity and sensitivity of US imaging and can be applied for various types of diseases, particularly tumors [5,6]. For this purpose, targeting strategies for microbubbles have been demonstrated [7,8,9,10,11]. Among these targeting strategies, antibodies have been used as a targeting moiety of microbubbles, and the avidin–biotin interaction has often been adopted to modify microbubbles with antibodies. However, avidin still has limitations in its usefulness because of the immunogenicity of streptavidin in humans [12]. Therefore, the clinical application of avidin–biotin interaction systems is difficult at present [13]. Consequently, a novel antibody modification is required for applications in clinical settings.

Polyethylene glycol (PEG)-modified liposomes have excellent biocompatibility, stability, and a long circulation time and have already been utilized in clinical applications [14,15]. These liposomes have also been widely used as carriers of drugs, antigens, and genes [14,15,16,17]. We recently reported that liposome-based nanobubbles (NBs) containing perfluorocarbon gas are suitable for US imaging and gene delivery [18,19,20,21,22,23].

**Figure 1:**
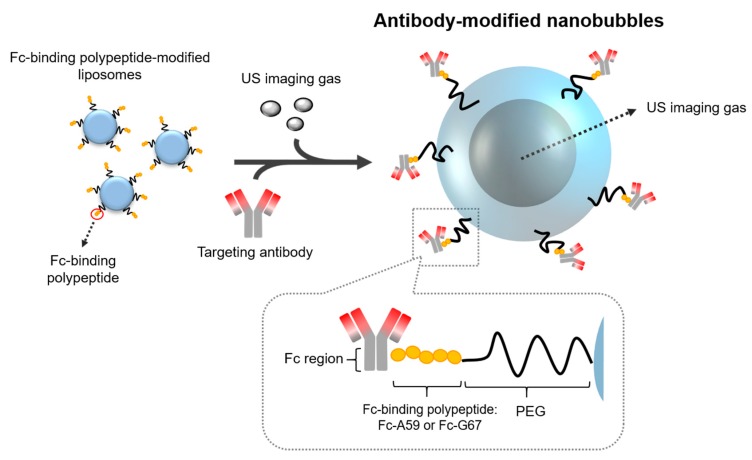
Schematic of the development of antibody-modified nanobubbles (NBs) using an Fc-binding polypeptide. PEG—polyethylene glycol; US—ultrasound.

In this study, to develop antibody-modified NBs for use in clinical settings, we focus on the fact that protein A/G binds strongly to immunoglobulin. Using the high binding affinity to the Fc region of an antibody, protein A/G has been widely used for purification [24]. Therefore, to develop antibody-modified NBs without the avidin–biotin interaction, we designed and purified Fc-binding polypeptides derived from protein A/G and developed antibody-modified NBs using these polypeptides (Figure 1: Schematic of the development of antibody-modified NBs using an Fc-binding polypeptide). As shown in Figure 1, we planned the development of the antibody modification method for NBs using an Fc-binding polypeptide. By using an Fc-binding polypeptide, antibodies can be modified on liposomes/NBs via the polypeptide. Because CD146 was expected to be a novel endothelial biomarker that acts as a co-receptor for vascular endothelial growth factor receptor-2 (VEGFR-2) in tumor angiogenesis [25], we chose CD146 as a tumor-targeting molecule for the ultrasound imaging agent. Therefore, we first prepared anti-CD146 antibody-modified NBs (m146-NBs). Next, to demonstrate their potential utility, we confirmed whether m146-NBs can specifically attach and function as a molecule-targeting US imaging agent in vitro and in vivo. To the best of our knowledge, this is the first report on the development of antibody-modified NBs using an Fc-binding polypeptide.

## 2. Materials and Methods

### 2.1. Production of Fc-Binding Polypeptides

In this study, we prepared two Fc-binding polypeptides (Fc-A59 and Fc-G67). Fc-A59 polypeptide was produced for modification of the anti-CD146 antibody based on previous reports [26]. Braisted et al. showed that the Z-domain of protein A, a three-helix bundle (59 residues), tightly binds to the Fc portion of IgG1 [26]. We designed a DNA fragment encoding the forward linker SGGSTS, the Z-domain of protein A, the backward linker ASTGS, and cysteine, synthesized by Fasmac Co., Ltd. (Kanagawa, Japan), which was then cloned into pGEX-6P-1 (GE Healthcare Bio-Sciences, Piscataway, NJ, USA). The Fc-G67 polypeptide was produced for modification of the anti-HER2 antibody (4D5-Fc) based on previous reports [27]. Briefly, Guss et al. reported that the C-terminal portion of protein G is responsible for the binding of IgG [27]. A 55-amino-acid sequence is repeated three times in the C1, C2, and C3 regions and binds to IgG. As described above, the DNA fragment encoding the spacer and C1 regions (67 residues) was synthesized and used for recombinant protein production. The recombinant proteins were produced in BL21 cells (Thermo Fisher Scientific, Waltham, MA, USA) using MagicMedia™ E. coli expression medium (Thermo Fisher Scientific, Waltham, MA, USA) and were purified with a glutathione sepharose column (GE Healthcare Bio-Sciences, Uppsala, Sweden).

### 2.2. Generation of an Antiserum for Mouse CD146 and 4D5-Fc Antibodies

Total RNA was isolated from the B16-F10 cells and used for cDNA synthesis. The cDNA segment encoding the mouse CD146 extracellular domain (amino acid sequence, 1–563) was amplified by PCR with KOD Plus DNA polymerase (TOYOBO, Osaka, Japan) using a primer set (Supplementary Table S1). The DNA fragment was verified using DNA sequencing analysis and was then cloned into the pEF4/V5-His vector. The recombinant mouse CD146 extracellular domain fused with 6xHis-tag (m146-His) was produced in HEK293 cells and purified from culture media using cobalt column chromatography. The recombinant protein was used for immunization in a rabbit model, and the specificity of the antiserum was verified using ELISA (data not shown). The IgG (m146 pAb) purified from the antiserum was used for further experiments.

The DNA encoding the anti-HER2 scFv fragment 4D5 [28,29] was synthesized by Fasmac Co., Ltd. (Kanagawa, Japan) and used as a template for PCR. The PCR product of 4D5 scFv was seamlessly joined to the DNA encoding the human laminin γ2 signal sequence by sequential PCR using a primer set (Supplementary Table S1), and restriction sites were introduced at the appropriate locations. The PCR products were subcloned into a human IgG1 Fc expression vector [30], and the recombinant antibody was prepared as described in our previous study [30]. The purified proteins were confirmed by SDS-PAGE using 5–20% gels under reducing conditions (Supplementary Figure S1).

### 2.3. Preparation of Antibody-Modified Liposomes and NBs

To prepare liposomes for the NBs, DSPC and DSPE-PEG_2000_-OMe were mixed at a molar ratio of 94:4. The liposomes were prepared by a reverse-phase evaporation method, as previously described [31,32]. Briefly, all lipids were dissolved in chloroform/diisopropyl ether at a ratio of 1:1 (*v*/*v*). PBS was added to the lipid solution, and the mixture was sonicated and then evaporated at 65 °C. The organic solvent was completely removed, and the size of the liposomes was adjusted to approximately 100–200 nm using extrusion equipment and a sizing filter (Nuclepore Track-Etch Membrane, 200 nm and 100 nm pore size, Whatman PLC, Kent, UK). After the sizing, the liposomes were passed through a sterile 0.45 μm syringe filter (Asahi Techno Glass Co., Chiba, Japan) for sterilization. For fluorescent labeling of the lipid membrane, 1,1-dioctadecyl-3,3,3,3-tetramethyl-indocarbocyanine perchlorate (DiI: 0.1 mol% of total lipids) was added. To modify the Fc-binding polypeptide in the liposomes, we used the post-insertion method [31]. Dried lipid films containing PEG_2000_–maleimide (2 mol%) were hydrated in PBS with gentle agitation and heating at 65 °C. An adequate amount of polypeptide (0.5 mol%) was added to the micelles in the presence of tris(2-carboxyethyl)phosphine hydrochloride (TCEP, final concentration: 20 mM), and the mixture was incubated for 6 h at room temperature (RT). To prepare polypeptide-modified liposomes, peptide-conjugated PEG micelles were mixed with the pre-formed liposomes for 1 h at 60 °C. To inactivate the free maleimide group, l-cysteine (final concentration: 0.1 mM) was added to the mixtures, followed by incubation for 15 min at RT. For the preparation of non-modified liposomes, 2 mol% of PEG_2000_–maleimide micelles without peptide were mixed with the pre-formed liposomes for 1 h at 60 °C. The resulting liposomes were passed through a Sephadex G-50 spin column to remove the excess TCEP and cysteine. Antibodies were added to the polypeptide-modified liposomes and incubated for 15 min at RT (4 μg antibody/100 μg lipid). The lipid concentration of the antibody-modified liposomes was measured using the phospholipid C test (Wako Pure Chemical Industries, Ltd., Osaka, Japan).

Each NB was prepared from antibody-modified liposomes and perfluoropropane gas (Takachiho Chemical Inc., Co., Ltd., Tokyo, Japan). As described in previous reports [19,23], 2 mL sterilized vials containing 0.8 mL of antibody-modified liposome suspension (lipid concentration: 1 mg/mL) were filled with perfluoropropane gas, capped, and then pressurized with 3 mL of perfluoropropane gas. The vials were placed in a bath-type sonicator (40 kHz, Bransonic 2800-J, Branson Ultrasonics Co., Danbury, CT, USA) for 2 min to produce NBs. The mean size of the NBs was determined via light scattering with a particle sizer (Nicomp 380ZLS, Santa Barbara, CA, USA). All lipids were purchased from NOF Corporation (Tokyo, Japan).

### 2.4. Cells and Tumor Model Mice

Human umbilical vein endothelial cells (HUVECs) were purchased from PromoCell (Heidelberg, Germany). The HUVECs were maintained in MCDB107 medium (Funakoshi, Tokyo, Japan), supplemented with 10% FBS, 20 ng/mL bovine brain extract (Kyokuto Pharmaceutical Industrial Co., Ltd., Tokyo, Japan), 10 ng/mL epidermal growth factor (EGF) (FUJIFILM Wako Pure Chemical Corporation, Osaka, Japan), and 50 μg/mL heparin (FUJIFILM Wako Pure Chemical Corporation, Osaka, Japan). All experiments were performed using HUVECs between passages 5 and 9. The HUVEC culture dishes were coated with type I collagen (Nitta Gelatin Inc., Osaka, Japan). SKOV3 cells were purchased from ATCC (Manassas, VA) and were maintained in McCoy’s 5 A medium containing 10% FBS. All cells were maintained at 37 °C in a humidified 5% CO_2_/95% air atmosphere.

Female KCN nu/nu mice (5–6 weeks old) were purchased from Japan SLC Inc. (Shizuoka, Japan). SKOV3 cells (5 × 10^6^ cells/mouse) were subcutaneously inoculated in the flanks of the mice. In vivo US imaging studies were performed when the tumors reached 100 mm^3^. Tumor volumes were calculated following the equation: volume (mm^3^) = (width × width) × (length) × 0.5. The animal use protocol and relevant experimental procedures were approved by the Committee of Animal Use and Welfare of Tokyo University of Pharmacy and Life Sciences (authorization number: P18-66).

### 2.5. Fluorescent Microscopic Analysis

HUVECs (3 × 10^4^ cells/well) were seeded in a 96-well plate and incubated overnight at 37 °C in 5% CO_2_. Then, 60 μL of NBs (Lipid concentration: 1 mg/mL) was mixed with the medium (360 μL) and added to the cells. The plates were sealed with sterile tape and inverted for 5 min. After incubation, the plates were reinverted for 5 min, and each well was washed with PBS twice to remove the non-adherent NBs. The cells were fixed with 4% paraformaldehyde, and the nuclei were counterstained with DAPI. The samples were then observed via fluorescent microscopy and analyzed using a BZ-X700 microscope (KEYENCE, Osaka, Japan).

### 2.6. In Vivo US Imaging Analysis

For in vivo US imaging, tumor-bearing mice (tumor size: approximately 100 mm^3^) were used (*n* = 3). The tumor-bearing mice were anesthetized and then intravenously injected with NBs as lipids at a dose of 200 μg/200 μL. The circles indicate the area of the tumor. US imaging was performed using an Aplio80 US diagnostic machine (Toshiba Medical Systems, Tokyo, Japan) and a 12-MHz wideband transducer with contrast harmonic imaging at a mechanical index of 0.25.

## 3. Results and Discussion

### 3.1. Design and Purification of Fc-Binding Polypeptide

**Figure 2:**
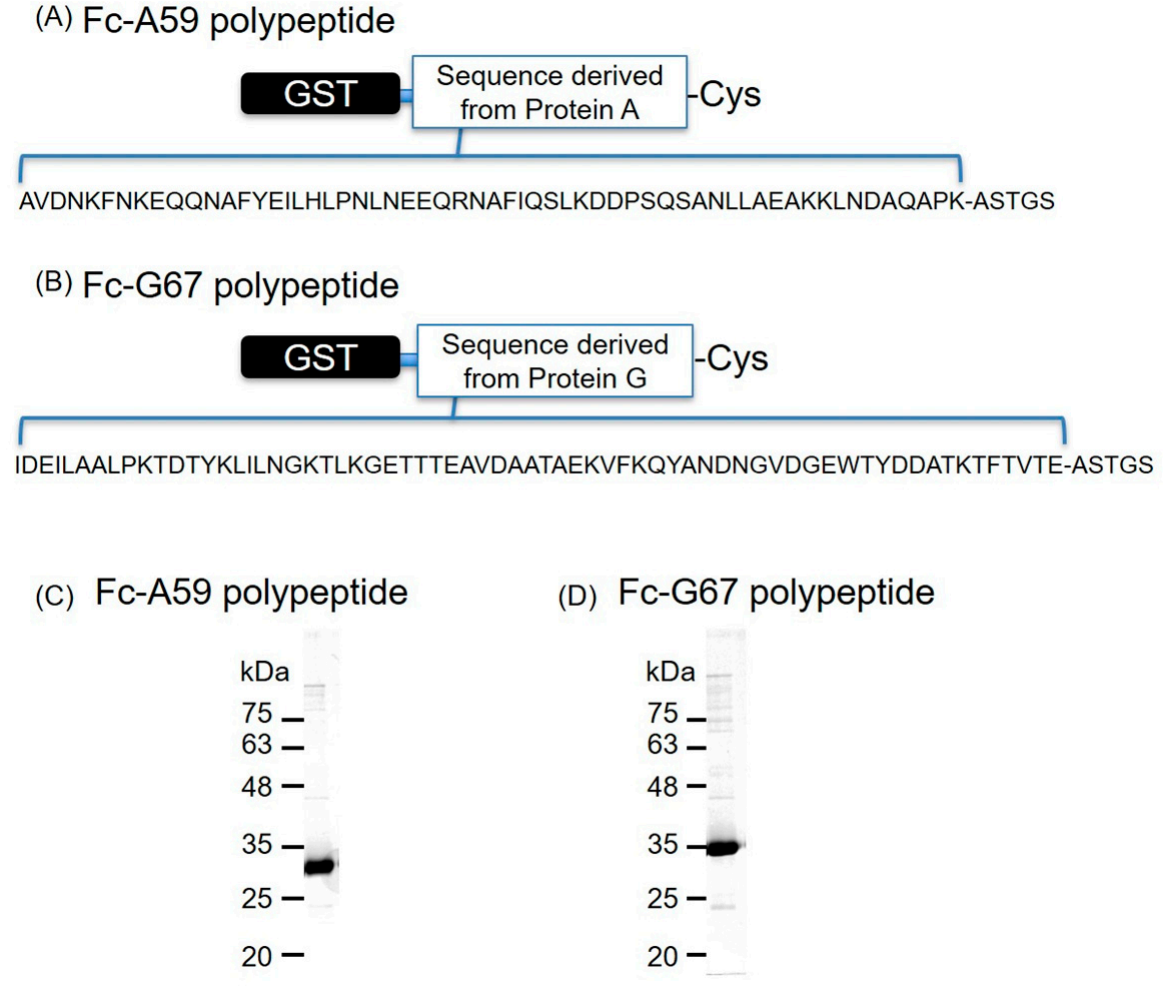
Design and purity of the Fc-binding polypeptide. (**A**,**B**) Schematic illustration and amino acid sequence of the Fc-binding polypeptides. (**C**,**D**) Confirmation of the purified polypeptides by SDS–PAGE.

Immunoglobulin binding proteins, such as protein A/G, have been widely used in the chromatographic purification of antibodies because of their high affinity to IgG (Kd = approximately 7 nM and 15 nM towards IgG, respectively) [33,34,35]. To develop antibody-modified NBs without the avidin–biotin interaction, we focused on the specific interaction between protein A/G and IgG. We first designed and purified the Fc-region-binding polypeptides derived from protein A/G, which were termed Fc-A59 polypeptide and Fc-G67 polypeptide (Figure 2A,B, respectively). SDS-PAGE analysis of these two purified polypeptides revealed a single protein band close to the expected molecular size (Fc-A59 polypeptide: 34.9 kDa, Fc-G67 polypeptide: 35.6 kDa) (Figure 2C,D). The binding affinity of the core sequence of the Fc-A59 polypeptide to the Fc region is extremely high (Kd = 10 nM) [26]. Indeed, the Fc-A59 and Fc-G67 polypeptides showed high binding affinity in ELISA (Supplementary Figure S2). Therefore, we used these polypeptides as an antibody modification tool for NBs.

### 3.2. Characteristics of Antibody-Modified Liposomes/NBs using Fc-Binding Polypeptide

We attempted to prepare antibody-modified liposomes and NBs. The average size of non-labeled liposomes (PEG-liposomes) was approximately 130 nm, while that of antibody-modified liposomes was approximately 170 nm. As shown in Supplementary Figure S3A, the size distributions of these liposomes were relatively narrow, and no aggregation was observed. As shown in Table 1 and Supplementary Figure S3B, the mean particle diameter of the non-labeled and antibody-modified NBs ranged from 500 to 700 nm, with a narrow distribution. The particle sizes were consistent with PEG-modified NBs, as shown in our previous reports [23,31]. This result suggests that even after antibody modification of the liposomes, NBs could still be prepared, resulting in an antibody-modified, nanosized lipid bubble.

### 3.3. Specific Attachment of Anti-CD146 Antibody-Modified NBs

**Figure 3:**
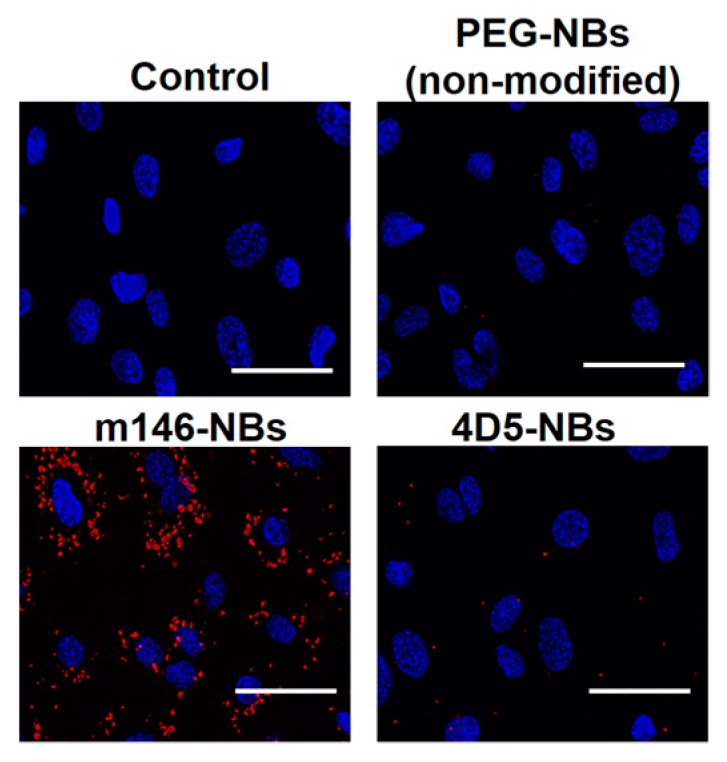
Specific attachment of anti-CD146 antibody-modified NBs to human umbilical vein endothelial cells (HUVECs). HUVECs were incubated with DiI-labeled NBs for 5 min. After incubation, the cells were washed and then treated with DAPI for nuclear staining. The treated cells were examined using a fluorescence microscope. Blue: DAPI fluorescence. Red: DiI fluorescence. Scale bar: 50 μm.

To examine the specific attachment of m146-NBs to HUVECs, which are known to express high levels of CD146 [36], the cell attachment of the NBs was observed by fluorescence microscopy. As shown in Figure 3, the m146-NBs exhibited a high level of attachment to the HUVECs, while attachment of non-modified NBs to the HUVECs was not observed. In this study, we used anti-HER2 antibody-modified NBs (4D5-Fc antibody-modified NBs: 4D5-NBs) as a control. Attachment of the 4D5-NBs to the HUVECs was rarely detected. However, the 4D5-NBs strongly attached to SKOV3 cells expressing high levels of HER2 [37], while the attachment of m146-NBs to SKOV3 cells was sparse (Supplementary Figure S4). These results demonstrate that the Fc-binding polypeptide works well as an antibody linker and can be applied to various antibodies with an Fc region for antibody modification. In addition, antibody-modified liposomes based on the Fc-binding polypeptide have a specific attachment ability, as observed for the antibody-modified NBs (Supplementary Figure S5). These results suggest that the antibody modification method using Fc-binding polypeptides may be a useful tool for antibody modification of nanoparticles.

### 3.4. In Vivo US Imaging

**Figure 4:**
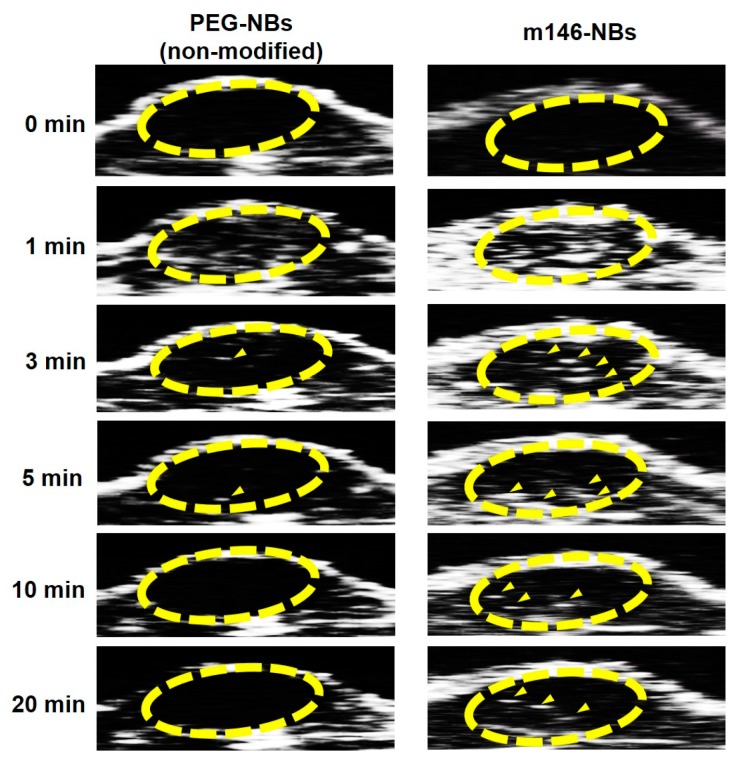
US imaging of tumor-bearing mice injected with NBs. Tumor-bearing mice (tumor size: approximately 100 mm^3^) were intravenously injected with NBs as lipids at a dose of 200 μg/200 μL. The circles show the area of the tumor, and the arrowheads indicate enhanced contrast images.

To evaluate the in vivo potential of the m146-NBs prepared by the antibody modification method using Fc-binding polypeptides, we performed US imaging in tumor-bearing mice. In this study, we obtained imaging data by using the contrast harmonic imaging technique, which exploits the nonlinear oscillations of microbubbles in contrast agents to detect microbubbles [38]. No animals suffered any injuries, such as burns, edema, or death, during this experiment. As shown in Figure 4, contrast images in the tumor area (yellow circle) were immediately detected as a white signal after the administration of non-modified NBs or m146-NBs. In particular, the m146-NBs showed strong signals at 1 min post-administration compared to the non-modified NBs. In addition, the contrast images for the m146-NB group were maintained for up to 20 min, in contrast to the observations for the non-modified group. CD146 has been reported as a novel endothelial biomarker and a target for tumor-related angiogenesis [25,36]. Therefore, this result suggests that m146-NBs may target tumor vessels, leading to longer US imaging. Furthermore, this result indicates that the antibodies on the NB surface were not displaced by IgG or other proteins in the blood. Therefore, the usefulness of our antibody modification method using Fc-binding polypeptides has been demonstrated. To perform antibody modification for microbubbles, maleimide–thiol chemistry is widely used [39,40,41], excluding the avidin–biotin method. In general, the maleimide–thiol reaction requires a long duration for conjugation (e.g., overnight or 24 h) [39,40,41]. In this report, it is suggested that the conjugation between the Fc-binding polypeptide and the Fc region of the antibody was completed within 15 min. Hence, the antibody modification method using Fc-binding polypeptides may be useful not only for avoiding immunogenicity but also for saving time in the conjugation step, if Fc-binding polypeptide-modified nanoparticles are prepared. Thus far, the combination of microbubbles and US exposure has been applied to deliver genes or nucleic acids (e.g., pDNA, siRNA, antisense oligonucleotides, shRNA, mRNA, and miRNA), and the administration of microbubbles loading genes or nucleic acids has enabled enhanced transfection efficiencies in targeted US sites in vivo [42,43,44,45]. We have also succeeded in developing cationic lipid-containing NBs loading pDNA, siRNA, and miRNA [19,20,21] and have demonstrated that the combination of NBs and US exposure can be applied as a useful gene or nucleic-acid delivery system in vivo [19,20,22]. Therefore, if cationic lipid-containing NBs are modified with a molecular/receptor-targeting antibody using the Fc-binding polypeptide, it is expected that therapeutic genes or nucleic acids could be delivered via a more restricted molecular/receptor targeting a disease site.

## 4. Conclusions

In this study, as an alternative to the avidin–biotin interaction in antibody modification, we developed antibody-modified NBs using Fc-binding polypeptides for US imaging. m146-NBs displayed specific attachment to HUVECs expressing CD146. Moreover, US imaging analysis demonstrated that the contrast images for the group injected with m146-NBs had a longer duration compared with those of the non-modified group, indicating that the antibodies on the NB surface were not displaced by IgG or other proteins in the blood. Thus, the modification method using Fc-binding polypeptides is suitable for providing an antibody linker. Therefore, this antibody-modified method using an Fc-binding polypeptide may be useful for developing next-generation antibody-modified US imaging agents. This is the first report on the development of antibody-modified NBs using Fc-binding polypeptides, rather than the avidin–biotin interaction.

## Figures and Tables

**Table 1:** Size of antibody-modified NBs and non-modified NBs.

NBs	Size (nm) ± SD
PEG-NBs (non-modified)	529.3 ± 66.9
m146-NBs (anti-mouse CD146 antibody-modified NBs)	614.2 ± 96.2
4D5-NBs (anti-HER2 antibody-modified NBs)	690.4 ± 106.1

## Supplementary Materials

The following are available online at https://www.mdpi.com/1999-4923/11/6/283/s1. Table S1: Primer sets. Figure S1: SDS-PAGE analysis of purified mouse CD146 antibody (A) and 4D5-Fc antibody (B). Figure S2: ELISA using the Fc-binding polypeptide demonstrates binding to the human IgG1 Fc. Figure S3: Size distribution of liposomes (A) and NBs (B). Figure S4: Specific attachment of anti-HER2 antibody-modified NBs to SKOV3 cells. Figure S5: Cellular uptake of antibody-modified liposomes (Lips) in HUVECs (A) and SKOV3 cells (B).
