# Supplementary material for: Development of Antibody-Modified Nanobubbles Using Fc-Region-Binding Polypeptides for Ultrasound Imaging
Source: Pharmaceutics. 2019 Jun 15;11(6):283. doi: 10.3390/pharmaceutics11060283 (PMC6631014; doi:10.3390/pharmaceutics11060283)
Supplement: Supplementary file 1 [file pharmaceutics-11-00283-s001.pdf]

pharmaceutics

Supplementary Materials: Development of Antibody-Modified Nanobubbles Using Fc-Region-Binding Polypeptides for Ultrasound Imaging

Nobuhito Hamano, Sho Kamoshida, Yamato Kikkawa, Yusuke Yano, Tomomi Kobayashi, Yoko Endo-Takahashi, Ryo Suzuki, Kazuo Maruyama, Yuji Ito, Motoyoshi Nomizu and Yoichi Negishi

Table S1. Primer sets.

rProtein	Primer	Sequence (5′-3′)
Mouse CD146	MsMCAM01	GGAATTCGCCACCATGGGGCTGCCCAAACTGGTGTGC
	MsMCAM02	CGTCTAGACCGACCACACCTTTGCTCTCTGGCTG
Signal sequence of human laminin γ2 chain	HG2SIG-03	CGCAATTGACCGCCATGCCTGCGCTCTG
	HG2SIG-06	AGACTCCACCAGCTGAACCTCCACTTCCCTCCTGGAGGTGGC
4D5 scFv	4D5-01	GCCACCTCCAGGAGGGAAGTGGAGGTTCAGCTGGTGGAGTCT
	4D5-02	CGCCTAGGCCTTTGATCTCCACCTTGGTACC

Experimental section

Production and purification methods were shown in Section 2.2. The purified proteins were confirmed by SDS-PAGE using 5–20% gels under reducing conditions.

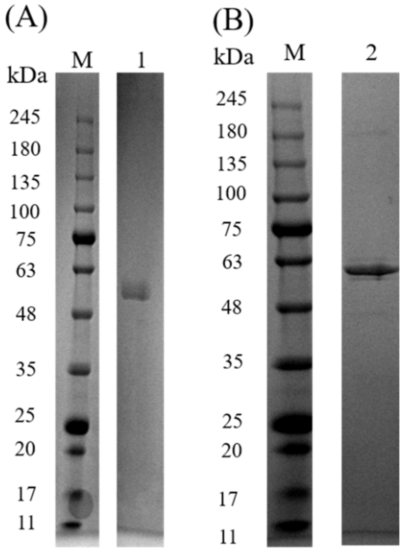

Figure S1. SDS-PAGE analysis of purified mouse CD146 antibody (**A**) and 4D5-Fc antibody (**B**). M: Molecular weight marker, 1: mouse anti-CD146 antibody (Reducing condition), 2: 4D5-Fc antibody (Reducing condition).

Ninety-six-well ELISA plates (Thermo Fisher Scientific) were coated with Fc-binding polypeptides (Fc-A59 and Fc-G67) and blocked with 1% BSA in PBS (−). Polypeptides were added and incubated at room temperature for 1 h. After washing with PBS-T, the bound polypeptides were detected using a biotinylated anti-human IgG1 Fc mAb (3 μg/mL). After further washing, the bound

antibodies were detected by addition of streptavidin-conjugated horseradish peroxidase, followed by addition of 0.4 mg/mL o-phenylenediamine and 0.01% H_2O_2. The absorbance was measured at 450 nm with a Multiskan GO microplate Spectrophotometer (Thermo Fisher Scientific).

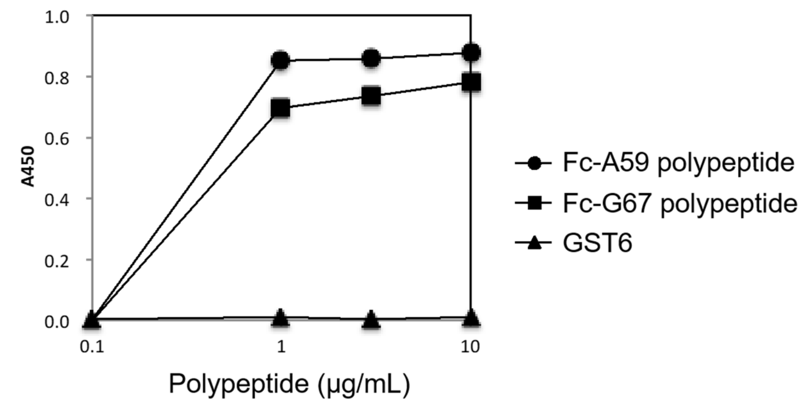

Figure S2. ELISA using the Fc-binding polypeptide demonstrates binding to the human IgG1 Fc.

Preparation methods of antibody-modified liposomes/NBs were shown in Section 2.3. The mean size and size distribution of the liposomes/NBs were determined via light scattering with a particle sizer (Nicomp 380ZLS, Santa Barbara, CA, USA).

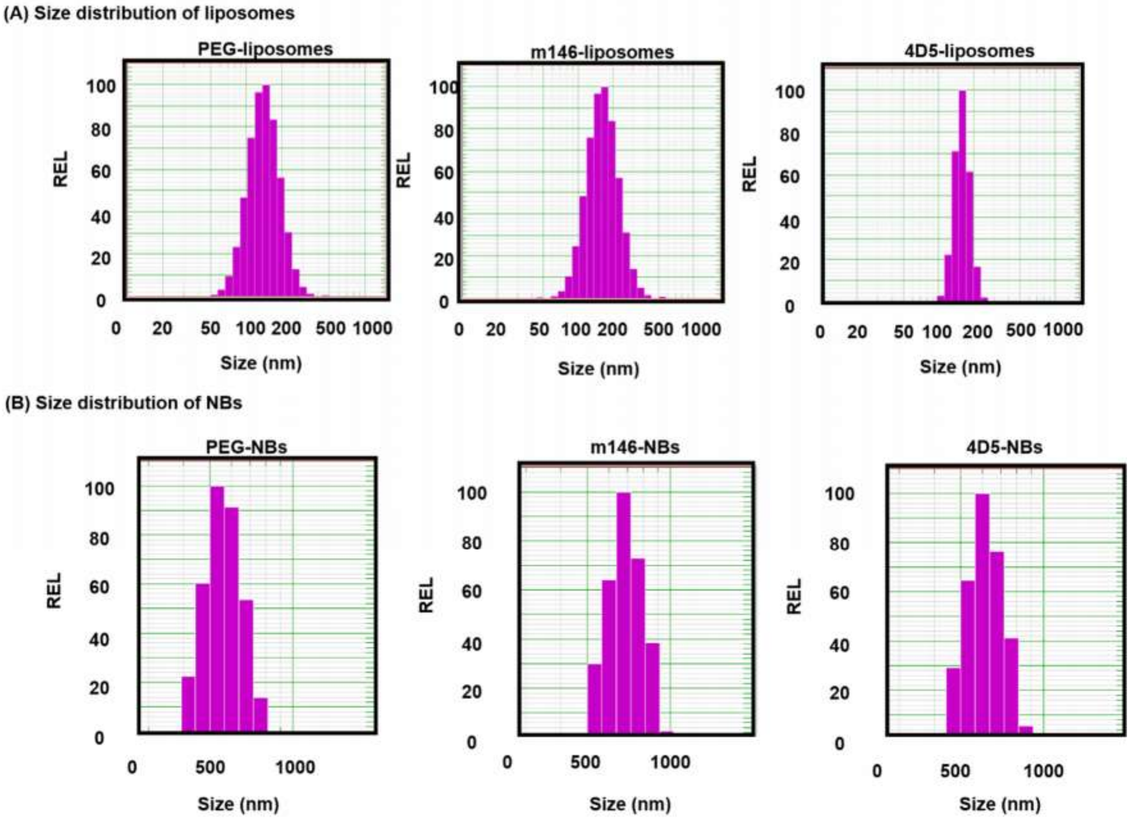

Figure S3. Size distribution of liposomes (**A**) and NBs (**B**).

The preparation of antibody-modified NBs is the same as in the manuscript. SKOV3 (3×10^4 cells/well) were seeded in the wells of a 96-well plate and incubated overnight at 37 °C in 5% CO_2. Then, 60 μL of NBs (Lipid concentration: 1 mg/mL) was mixed with the medium (360 μL) and added to the cells. The plates were sealed with sterile tape and inverted for 5 min. After incubation, the plates were reinverted for 5 min, and each well was washed with PBS twice to remove the non-adherent NBs. The cells were fixed with 4% paraformaldehyde, and the nuclei were counterstained

with DAPI. The samples were then observed via fluorescent microscopy and analyzed using a BZ-X700 microscope (KEYENCE, Japan).

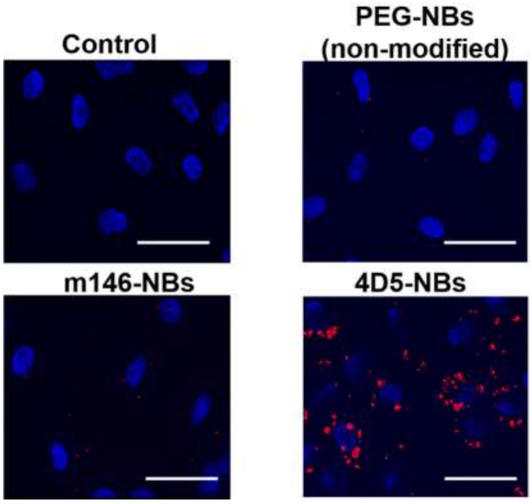

Figure 4. Specific attachment of anti-HER2 antibody-modified NBs to SKOV3. SKOV3 were incubated with DiI-labeled NBs for 5 min. After incubation, the cells were washed and treated with DAPI for nuclear staining. The treated cells were examined using a fluorescence microscope. Blue: Fluorescence of DAPI, Red: Fluorescence of DiI. Scale bars represent 50 µm.

The preparation of polypeptide-modified Lips is the same as in the manuscript. Antibodies were added into polypeptide-modified liposomes and incubated for 15 min at RT (2 µg antibody/100 µg lipid). The lipid concentration of antibody-modified liposomes was measured using the Phospholipid C test (Wako Pure Chemical Industries, Ltd., Osaka, Japan). SKOV3 or HUVEC (3×10^4 cells/well) were seeded in the wells of a 96-well plate and incubated overnight at 37°C in 5% CO_2. 100 µg of Lips was mixed with the medium and added to the cells. The plates were incubated for 60 min at 37 °C in 5% CO_2. After incubation, each well was washed with PBS twice to remove free Lips. Cells were fixed by 4% paraformaldehyde, and the nucleus was counterstained with DAPI. The samples were fluorescently observed and analyzed using a BZ-X700 microscope (KEYENCE, Japan).

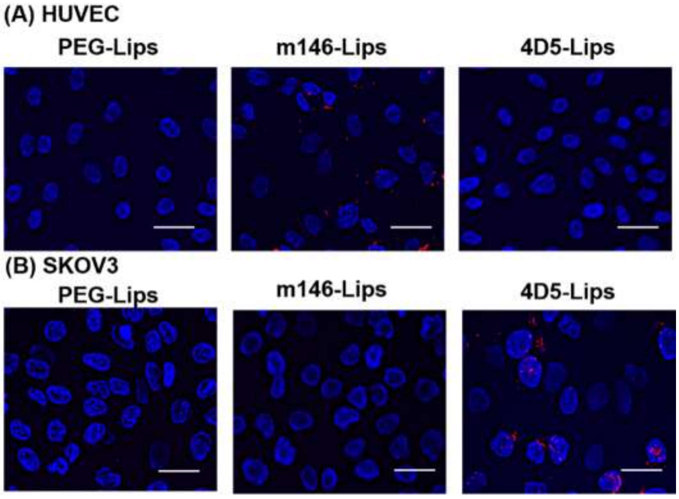

Figure 5. Cellular uptake of antibody-modified liposomes (Lips) into HUVEC (**A**) and SKOV3 (**B**). Cells were incubated with DiI-labeled lips for 60 min. After incubation, the cells were washed and treated with DAPI for nuclear staining. The treated cells were examined using a fluorescence microscope. Blue: Fluorescence of DAPI, Red: Fluorescence of DiI. Scale bars represent 30 µm.
